# Clinical Evaluation of Corridor Disease in *Bos indicus* (Boran) Cattle Naturally Infected With Buffalo-Derived *Theileria parva*

**DOI:** 10.3389/fvets.2021.731238

**Published:** 2021-09-29

**Authors:** Elizabeth A. J. Cook, Tatjana Sitt, E. Jane Poole, Gideon Ndambuki, Stephen Mwaura, Maurine C. Chepkwony, Perle Latre de Late, Antoinette A. Miyunga, Richard van Aardt, Giles Prettejohn, David Wragg, James G. D. Prendergast, W. Ivan Morrison, Philip Toye

**Affiliations:** ^1^Livestock Genetics, International Livestock Research Institute (ILRI), Nairobi, Kenya; ^2^Centre for Tropical Livestock Genetics and Health (CTLGH), International Livestock Research Insitute (ILRI) Kenya, Nairobi, Kenya; ^3^Ol Pejeta Conservancy, Nanyuki, Kenya; ^4^The Roslin Institute, University of Edinburgh, Easter Bush Campus, Roslin, United Kingdom; ^5^Centre for Tropical Livestock Genetics and Health (CTLGH), Easter Bush Campus, Roslin, United Kingdom

**Keywords:** *Theileria parva*, corridor disease, East Coast fever (ECF), buffalo, cattle

## Abstract

Corridor disease (CD) is a fatal condition of cattle caused by buffalo-derived *Theileria parva*. Unlike the related condition, East Coast fever, which results from infection with cattle-derived *T. parva*, CD has not been extensively studied. We describe in detail the clinical and laboratory findings in cattle naturally infected with buffalo-derived *T. parva*. Forty-six cattle were exposed to buffalo-derived *T. parva* under field conditions at the Ol Pejeta Conservancy, Kenya, between 2013 and 2018. The first signs of disease observed in all animals were nasal discharge (mean day of onset was 9 days post-exposure), enlarged lymph nodes (10 days post-exposure), and pyrexia (13.7 days post-exposure). Coughing and labored breathing were observed in more than 50% of animals (14 days post-exposure). Less commonly observed signs, corneal edema (22%) and diarrhea (11%), were observed later in the disease progression (19 days post-exposure). All infections were considered clinically severe, and 42 animals succumbed to infection. The mean time to death across all studies was 18.4 days. The mean time from onset of clinical signs to death was 9 days and from pyrexia to death was 4.8 days, indicating a relatively short duration of clinical illness. There were significant relationships between days to death and the days to first temperature (chi^2^ = 4.00, *p* = 0.046), and days to peak temperature (chi^2^ = 25.81, *p* = 0.001), animals with earlier onset pyrexia died sooner. These clinical indicators may be useful for assessing the severity of disease in the future. All infections were confirmed by the presence of macroschizonts in lymph node biopsies (mean time to parasitosis was 11 days). Piroplasms were detected in the blood of two animals (4%) and 20 (43%) animals seroconverted. In this study, we demonstrate the successful approach to an experimental field study for CD in cattle. We also describe the clinical progression of CD in naturally infected cattle, including the onset and severity of clinical signs and pathology. Laboratory diagnoses based on examination of blood samples are unreliable, and alternatives may not be available to cattle keepers. The rapid development of CD requires recognition of the clinical signs, which may be useful for early diagnosis of the disease and effective intervention for affected animals.

## Introduction

*Theileria parva*, an apicomplexan protozoal parasite, is the causative agent of both East Coast fever (ECF) and corridor disease (CD) in cattle. ECF is caused by cattle-derived *T. parva*, and CD is caused by buffalo-derived *T. parva*. Historically, it was considered that the two diseases were caused by two separate *Theileria* parasites, *T. parva parva* and *T. parva lawrencei* (1). More recently, however, it was discerned through genetic analysis that the two diseases are caused by the same parasite, *T. parva*, with differences in pathogenicity and virulence (2).

A great deal of research effort has been focused on understanding and controlling *T. parva* infections since the first recorded significant loss of cattle in the 1890s (2). A large amount of research on *T. parva* infection has been conducted in controlled challenge experiments (3, 4). The majority of research has focused on ECF, including, but not limited to, identifying parasite antigens recognized by the bovine immune system, assessment of the genetic diversity of the parasite, and the development and testing of new subunit vaccines (5–8). In contrast, detailed research of CD is limited, and detailed descriptions regarding the clinical manifestations of CD and experimental infections have been incomplete. The first description of the clinical presentation of CD was published in 1955 but was limited to a list of the clinical signs (9). A comparison of the disease progression between ECF and CD was conducted in 1980 with a needle challenge experiment using *T. parva* stabilates from cattle and buffalo, respectively (10). A more recent publication described CD outbreaks in South Africa at the wildlife/livestock interface but did not give a detailed description of the clinical disease in the affected animals (11).

The clinical presentation of both ECF and CD includes enlarged lymph nodes, pyrexia, lacrimation, nasal discharge, anorexia, teeth grinding, loss of body condition, diarrhea, labored breathing and coughing, recumbency, and death (9, 12, 13). CD has previously been distinguished from ECF by lower parasite levels and rare detection of piroplasms (1, 9).

Diagnosis of ECF and CD in the field is predominantly clinical, although there are confirmatory tests available. The main diagnostic test available to researchers and cattle keepers is the detection of macroschizonts in Giemsa stained lymph node aspirates, although serological and molecular assays are available (14). There are currently no diagnostic methods to distinguish between infection with ECF and CD.

Control methods include reducing exposure to the parasite by controlling the tick vector through the use of acaricides and the use of a vaccination method known as the infection and treatment method (ITM) (15, 16). Immunization is achieved by administering a ground homogenate of infected sporozoite-containing ticks while simultaneously preventing the progression of the disease by administering a dose of long-acting oxytetracycline (16). The efficacy of the ITM in preventing CD is variable; ITM is effective in Tanzania against disease caused by buffalo-derived *T. parva* but not in Kenya (17–20). A deeper understanding of CD is needed, particularly with regard to distinguishing CD and improving its control in light of the reduced efficacy of the vaccine (17).

Most research describing the clinical signs of *T. parva* infection (both ECF and CD) has been conducted with experimentally infected cattle achieved by injecting cattle with *T. parva* stabilate or applying infected ticks (3, 10). The purpose of this paper is to describe in detail the clinical manifestations and disease progression of CD in a naturally infected Boran calf population, thus contributing to a more thorough and detailed description of CD than has historically been described. In addition, we describe the approach to conducting a clinical field study, including methods for ensuring naïveté and exposure in study animals, measuring clinical parameters in the field, and confirmation of disease and requirements of the study site.

## Materials and Methods

### Animal Care and Use

The study protocols were approved by the International Livestock Research Institute (ILRI)'s Institutional Animal Care and Use Committee (references 2013-03, 2014-32, 2015-29, 2017-02, and 2018-10). The humane endpoint was defined as 10 days of elevated temperature or if the animal developed severe signs of disease, particularly recumbency and anorexia, as assessed by two veterinarians. The animals were treated or euthanized depending on the severity of the disease.

### Study Site

**Figure 1:**
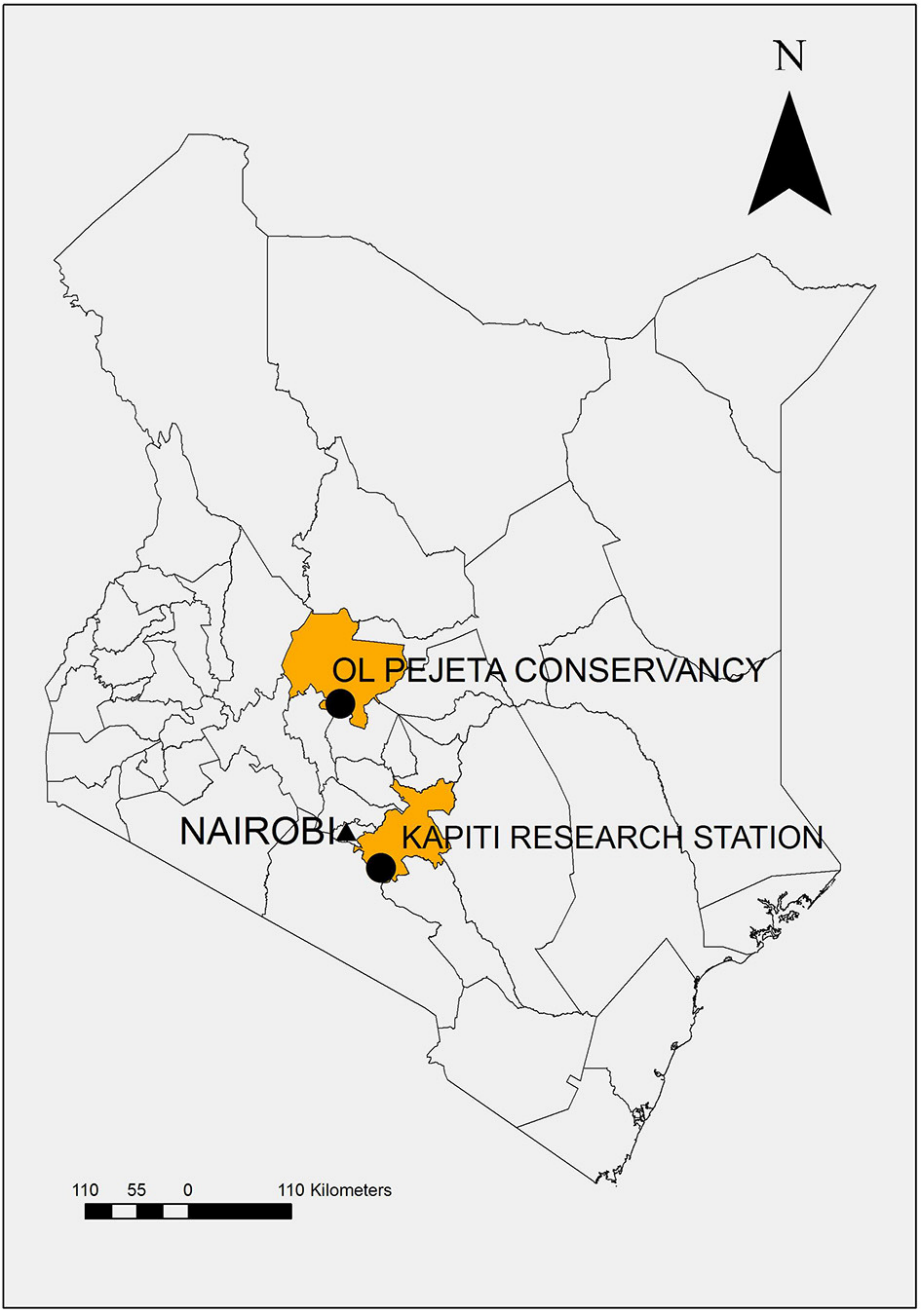
Map of Kenya demonstrating study location, Ol Pejeta Conservancy, Laikipia County, and source of study animals, Kapiti Research Station, Machakos County.

The field studies were conducted at the Ol Pejeta Conservancy in Laikipia County, Kenya. Ol Pejeta is considered endemic for CD (Figure 1) (17). The field study sites were located at 00°03.052′S, 36°52.302′E; altitude 1,784 m (2013) and 00°00.971'N, 36°55.171'E; altitude 1,797 m (2014–2018), which is 9 km northeast of the 2013 site. Both sites are routinely grazed by buffalo and other wildlife but not cattle.

To determine the presence of infected ticks in the study area, drag sampling with cloth was used to collect ticks from pasture. *Rhipicephalus appendiculatus* ticks were identified morphologically and removed from the cloth with forceps before being placed in breathable containers with moisture for transportation to ILRI for further analysis. The tick salivary glands were removed and examined by microscopy for the presence of infected acini. DNA was extracted from the glands and analyzed by polymerase chain reaction (PCR) involving amplification of the *T. parva* p67 gene (21).

### Study Animals

The study animals were part of five experimental field studies investigating CD in Boran cattle conducted between 2013 and 2018. The first study in 2013 was an assessment of the Muguga cocktail live sporozoite vaccine and has been described previously (17). The subsequent studies (2014–2018) investigated tolerance to CD in a group of genetically related, unvaccinated cattle compared with unrelated controls. The results regarding the genetically related cattle will be reported elsewhere.

Only unvaccinated cattle (2013) and control cattle genetically unrelated to the animals being studied for tolerance to CD (2014–2018) were used to conduct the analyses in this paper to exclude any influence of immunity or pedigree. The relatedness of cattle was determined by pedigree records and confirmed by genotyping (22).

Cattle, previously unexposed to *T. parva*, were purchased from ILRI's Kapiti Research Station (Machakos, Kenya) and transported to Ol Pejeta Conservancy (Laikipia, Kenya) (Figure 1). Forty-six cattle were observed in the five studies 2013 (*n* = 9), 2014 (*n* = 14), 2015 (*n* = 10), 2017 (*n* = 7), and 2018 (*n* = 6). Cattle were aged between 8 and 18 months, and the majority of the study cattle were steers or bulls (*n* = 33) except for 13 heifers (2015 *n* = 5, 2017 *n* = 5, and 2018 *n* = 3).

All cattle tested negative for *T. parva* exposure or presence *via* enzyme-linked immunosorbent assay (ELISA) for antibodies to the polymorphic immunodominant molecule, a *T. parva* antigen expressed by sporozoites and schizonts (23) (all studies) and nested PCR targeting the p104 gene (24) (2013, 2017, 2018) before transport to the study site, confirming freedom from disease (Supplementary Table 1). All study cattle were in good health before transport to the field study site. Preventive medicine measures included: vaccination against foot-and-mouth disease (Kenya Veterinary Vaccines Production Institute) at least 1 month before shipment, treatment with an acaricide (DuoDip, Norbrook, Nairobi, Kenya) 5 days before shipment, and a pre-shipment treatment of multivitamins (Norbrook, Nairobi, Kenya).

### Field Study and Clinical Health Assessment

At the study site, cattle were housed in a fenced yard at night and allowed to graze freely during the day. Ticks attached naturally onto the cattle, and for ethical reasons, cattle were treated with acaricide when a heavy tick burden was observed after 10–17 days of the field exposure. After the initial treatment, cattle were treated with acaricide every 3–7 days to inhibit excessive tick attachment.

Animal health and clinical observations were assessed daily by at least two researchers before the cattle were released from the pens for grazing. In addition, the herders who were with the cattle around the clock reported any clinical observations such as anorexia, coughing, diarrhea, weakness, or prolonged periods of recumbency. Temperatures and clinical signs of disease (corneal opacity, lacrimation, nasal discharge, cough, labored breathing, lymph node swelling, body condition, and anorexia) were included in the health assessment. Any other abnormal parameters including, but not limited to, stool consistency, ventral edema (bottle jaw), teeth grinding, and neurological symptoms were also recorded.

Rectal temperatures were measured daily, and pyrexia was defined as core body temperature ≥ 39.5°C (3). Coughing was recorded if an animal coughed more than once either during the observation period or throughout the day, labored breathing was defined as difficulty breathing with or without wheezing during the morning observation period, lymph node swelling was determined *via* palpation and visual assessment, poor body condition was agreed upon by two researchers during the observation period, and anorexia was defined as not eating for at least 1 day or eating extremely little.

### Field Sampling and Laboratory Analysis

Blood was collected into ethylenediaminetetraacetic acid vacutainers (BD™) from the jugular veins of animals every day (2015, 2017, and 2018) or every 3–4 days before disease manifestation and every 1–2 days during acute disease, post-initial pyrexia (2013, 2014). Blood was collected into serum vacutainers (BD™) every other day (2013) and every 5 days (2014–2018).

Filled ethylenediaminetetraacetic acid and serum vacutainers were kept cool in ice boxes with frozen ice packs in the field before transport to the laboratory for analysis. Whole blood was used to conduct white blood cell (WBC) counts, packed cell volume (PCV), and aliquots frozen in liquid nitrogen for nested p104 PCR analysis for confirmation of parasite infection, as described previously (24). Serum was separated by centrifugation at 3,000 rpm for 20 min and two aliquots frozen.

WBC counts were conducted using a hemocytometer throughout the studies in 2015 and 2017, every second day in 2014, and in the last 10 days of the 2013 study. PCV was measured using a hematocrit reader with a capillary tube, centrifuged at 3,000 rpm for 6 min throughout the 2015, 2017, and 2018 studies and every 3–4 days before disease manifestation and every 1–2 days during acute disease (2013, 2014). Total plasma protein (TPP) was estimated every day in the 2015, 2017, and 2018 studies using a refractometer on the supernatant from the PCV capillary tubes.

Needle biopsies were taken from parotid and pre-scapular lymph nodes every day (2015, 2017, and 2018) or every second day (2013 and 2014) from the first day of swelling to the end of the study or death. In the initial studies, the purpose of the sampling was to detect macroschizonts and confirm infection; in subsequent studies, a more systematic approach was taken to estimate the day of infection. Lymph nodes were sampled alternately on the left and right sides of the animal to minimize damage. If a lymph node aspirate was negative three samples in a row, after being macroschizont-positive, the animal was no longer sampled.

### Enzyme-Linked Immunosorbent Assay

Serum samples collected every 5 days after exposure were screened for antibodies to *T. parva* by ELISA as previously described (23). In brief, microtiter plates were coated with a recombinant (glutathione-S-transferase) version of the polymorphic immunodominant molecule antigen. Test serum samples were added in duplicate at a dilution of 1:200, and bound antibodies were detected with a horseradish peroxidase-linked mouse monoclonal antibody specific for bovine IgG1. After the addition of the substrate hydrogen peroxide and 2,2'-azino-di-[3-ethyl-benzothiazoline-6-sulphonic acid, the mean OD_405_ of the two wells was recorded. Interassay variation was controlled by analyzing four control sera—a strong positive, a moderately positive, a weak positive, and a negative sample. The positive sera were obtained from an animal infected with *T. parva* Muguga stabilate 3087 (25). Each control serum was added in quadruplicate, and the resultant OD_405_ values had to fall within predetermined limits for the assay to be valid. The percentage positivity (PP) relative to the strong positive sample was determined for each sample using the formula: PP = (mean OD_sample_/mean OD_strongpositive_) × 100. Samples with PP of 20 or greater were considered positive. The test does not differentiate between ECF or CD infection.

### Polymerase Chain Reaction

The p67 PCR was performed as previously described (21). The master mix consisted of 5 μl of OneTaq® 2 × Master Mix (New England Biolabs), 0.2 μl each primer (Table 1), 3.1 μl water, and 1.5 μl of DNA. The cycling conditions were 95°C for 5 min, 30 cycles of 95°C for 1 min, 56°C for 1 min, 72°C for 1 min, then 72°C for 9 min and a hold at 4°C.

**Table 1:** Primers used to detect *T. parva* genes p67 and p104.

**Genes**		**Sequence 5^**′**^-3^**′**^**	**Size (bp)**
p67 PCR (21)	Foward	CGACACTGAACGATGCAAATA	2200
	Reverse	GAGTTATTGTTAGTGGACGAT	
p104 PCR (outer) (24)	Forward	ATTTAAGGAACCTGACGTGACTGC	496
	Reverse	TAAGATGCCGACTATTAATGACACC	
P104 PCR (inner)	Forward	GGCCAAGGTCTCCTTCAGAATACG	277
	Reverse	TGGGTGTGTTTCCTCGTCATCTGC	

The nested p104 PCR was performed as previously described (24). The master mix consisted of 5 μl of DreamTaq PCR Master Mix (2 × ) (Thermo Scientific), 0.2 μl each primer (Table 1), 3.6-μl water, and 1 μl of DNA (outer primer reaction) or 1-μl first reaction PCR product (inner primer reaction). The cycling conditions were 95°C for 5 min, then 30 cycles of 95°C for 1 min, 60°C for 1 min, 72°C for 1 min (outer reactions) or 30 cycles of 95°C for 1 min, 55°C for 1 min, 72°C for 1 min (inner reaction), then 72°C for 9 min and a hold at 10°C.

Negative (no DNA) and positive controls were included in each PCR. The positive control was DNA extracted from blood drawn from an animal infected with *T. parva* Muguga stabilate 3087 (25). PCR products underwent electrophoresis through a 1.5% agarose gel with GelRed (Biotium) and were visualized under ultraviolet light.

### Microscopy

Lymph node and blood smears were stained with Giemsa and examined for the presence of parasites and hyperplastic cells. The level of macroschizonts was recorded using the following scores: Ma+, <1 parasite per field; Ma++, 1–2 parasites per field; and Ma+++, >2 parasites per field under 500 × objective (3).

### Postmortem Procedure

Cattle that succumbed to infection or were euthanized were necropsied in the field. The animal was positioned in left lateral recumbency and a skin incision made from the chin to the perineum. The skin was reflected and the right limbs abducted. The abdominal muscles were reflected by cutting along the costal arch. Rib cutters were used to remove the ribs by cutting along the sternum and close to the vertebrae. The organs were observed *in situ* and then examined for specific gross lesions associated with CD (4, 26). Digital photographs were taken of the animal and the organs. The trachea was examined for the presence of froth. Lungs were examined for evidence of pulmonary edema, and lymph nodes (pre-scapular and parotid) were examined for edema and hemorrhage. Lesions were recorded as mild, marked, or absent.

### Confirmation of Infection

Infection with *T. parva* was confirmed using multiple methods, microscopy, PCR (24), ELISA (23), and postmortem findings (13). Impression smears of organs (lung, liver, kidney, spleen, parotid, and pre-scapular lymph nodes) were collected postmortem to confirm parasitosis (13).

### Descriptive Statistics

Several continuous parameters were assessed (3). Lymph node infection parameters included the first day of lymph node positivity (either parotid or pre-scapular as sampling was dependent on lymph node swelling) and average intensity of positivity across all lymph node samples. Infection intensity is based on the number of schizonts seen *via* microscopy in the lymph node aspirate samples scored as 1–3 (Ma+ =1, Ma++ =2, and Ma+++ =3) and divided by the number of days of schizont detection (3). The duration was measured as days between infection and death (non-survivors). Pyrexia parameters included the first day of pyrexia, the average intensity of the pyrexia over time, the duration of pyrexia per animal, the days to a peak temperature, and the peak value. Temperature intensity was the sum of the difference from normal (39.4°C), divided by the number of days of pyrexia over 39.4°C. Piroplasm parameters included the day of first piroplasm detection, intensity (presented as the number of piroplasm per 1,000 RBC counted), and duration.

The agreement between studies was measured using analysis of variance, in the R environment for statistical computing, version 3.4.0 (http://cran.r-project.org/), to compare the means for day to death, the first day of lymph node positivity, the first day of pyrexia, days to peak temperature, peak temperature value, temperature intensity, and PCV. The relationship between the day of death and the described variables was compared using mixed-effects linear regression models in the *lme4* package in R (27) with the study entered as a random effect. Likelihood ratio tests of the full model with the covariate in question against the model without the covariate were used to calculate *p*-values.

Non-continuous clinical parameters were recorded as present or absent and included corneal opacity, lacrimation, nasal discharge, cough, labored breathing, lymph node swelling, loss of condition, anorexia, neurological signs, teeth grinding, diarrhea, bottle jaw, and urticaria.

## Results

All the cattle (*n* = 46) developed clinical signs consistent with CD infection. The mean values of the continuous clinical parameters for each study are presented in Table 2. A limited number of the results for the 2013 study have been published elsewhere (17) and are included in Table 2 here for comparison. Key results for each parameter are presented later.

**Table 2:** Mean values for clinical variables measured during five trials *N* = 9 (2013), 14 (2014), 10 (2015), 7 (2017), and 6 (2018).

**Variable**	**2013 *n* = 9**	**2014*n* = 14**	**2015 *n* = 10**	**2017*n* = 7**	**2018 *n* = 6**	**Mean*n* = 46**	**Total range**	**Number of animals with data points**
Mean time to death (days)	21.4	16.2	18.9	17.3	18.8	18.4	15–27	42
Macroschizont—first (days post-challenge)	NE	NE	10.8	11.9	10.2	11.0	7–14	23
Macroschizont intensity (score 1–3)	NE	NE	1.59	1.49	1.53	1.55	1–2.4	23
Macroschizont duration (onset to death)	NE	NE	8.10	5.67	9.25	7.6	4–12	23
Pyrexia—first (days post challenge)	14.7	14.3	12.8	12.6	13.3	13.7	10–19	46
Pyrexia intensity (score 1–3)	1.15	1.62	0.99	1.06	1.03	1.23	0.4–3.0	46
Time from pyrexia to death	6.8	2.0	6.1	4.5	6.3	4.76	1.0–13	42
Pyrexia—peak (°C)	41.3	41.7	41.2	41.2	41.3	41.4	40.2–43.3	46
Pyrexia—peak range (°C)	40.5–41.7	41.1–43.3	40.6–41.9	40.6–42.0	40.2–41.8	NA	NA	46
Pyrexia—peak time (days)	17.6	15.3	16.1	14.9	15.8	15.9	13–22	46
WBC ( × 10^6^/L) (average last 4 days)	NE	NE	6200	6700	NE	6400	3,500–9,000	17
PCV (Final PCV/Day 0 PCV × 100)	85%	91%	92%	90%	78%	88%	64–117%	46
Protein (Final Protein/Day 0 Protein × 100)	NE	NE	74%	72%	63%	70%	57–83%	23

*NE, not examined; NA, not applicable*.

### Survival

**Figure 2:**
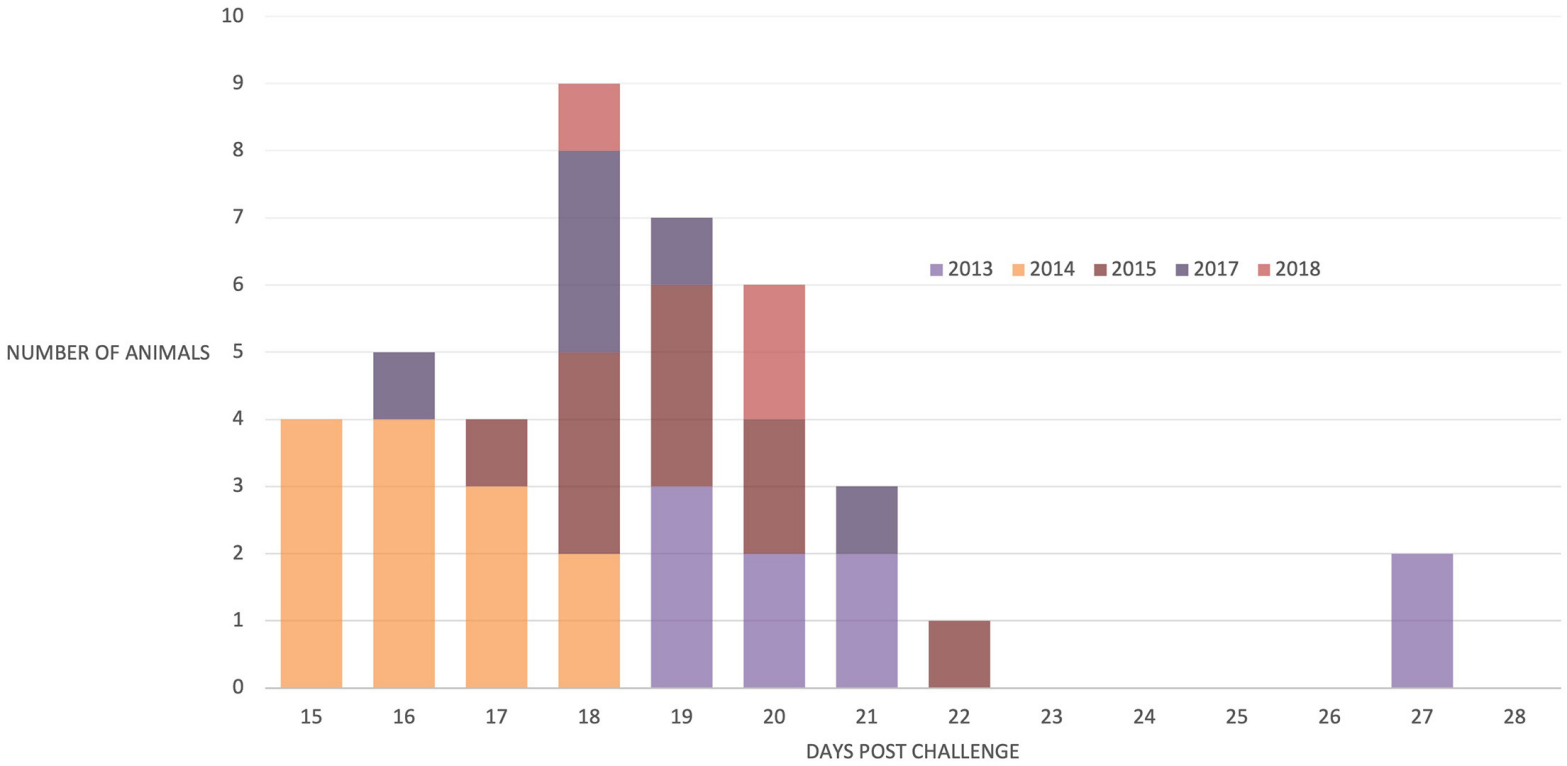
Number of animals that succumbed to disease post-natural challenge in all five trials. *N* = 9 (2013), 13 (2014), 10 (2015), 6 (2017), and 4 (2018).

Forty-two (91%) of the 46 animals succumbed to CD, indicating that the animals were exposed to about a lethal dose of 100% (LD_100_) in each study. Of the 42 animals that succumbed, 30 died, 5 were euthanized, and 7 were treated; all 42 animals were *in extremis* and considered to have died from CD for the purposes of this study. One animal survived in each of the 2014 and 2017 studies and two in the 2018 study. There was a significant difference in the mean time to death between studies [*F*_(4, 37)_ = 10.16, *p* < 0.001]. However, within each study, most animals succumbed over a relatively short interval of 3–5 days (Figure 2), suggesting that the animals were uniformly exposed after arrival at the study site. In this regard, 19 *R. appendiculatus* ticks (10 females and 9 males) were collected from the pasture at the study site and examined for the presence of *T. parva*. Infected acini were observed in six ticks, and these and a further two ticks were shown to be positive by both p67 and 18S RNA gene amplification by PCR; an overall infection rate was 42%. The abundance calculated by dividing the number of infected acini (113) by the total number of ticks (19) was 5.95. The intensity calculated by dividing the number of infected acini (113) by the number of infected ticks (6) was estimated to be 18.8.

### Lymph Node Parasitosis and Parasitemia

**Figure 3:**
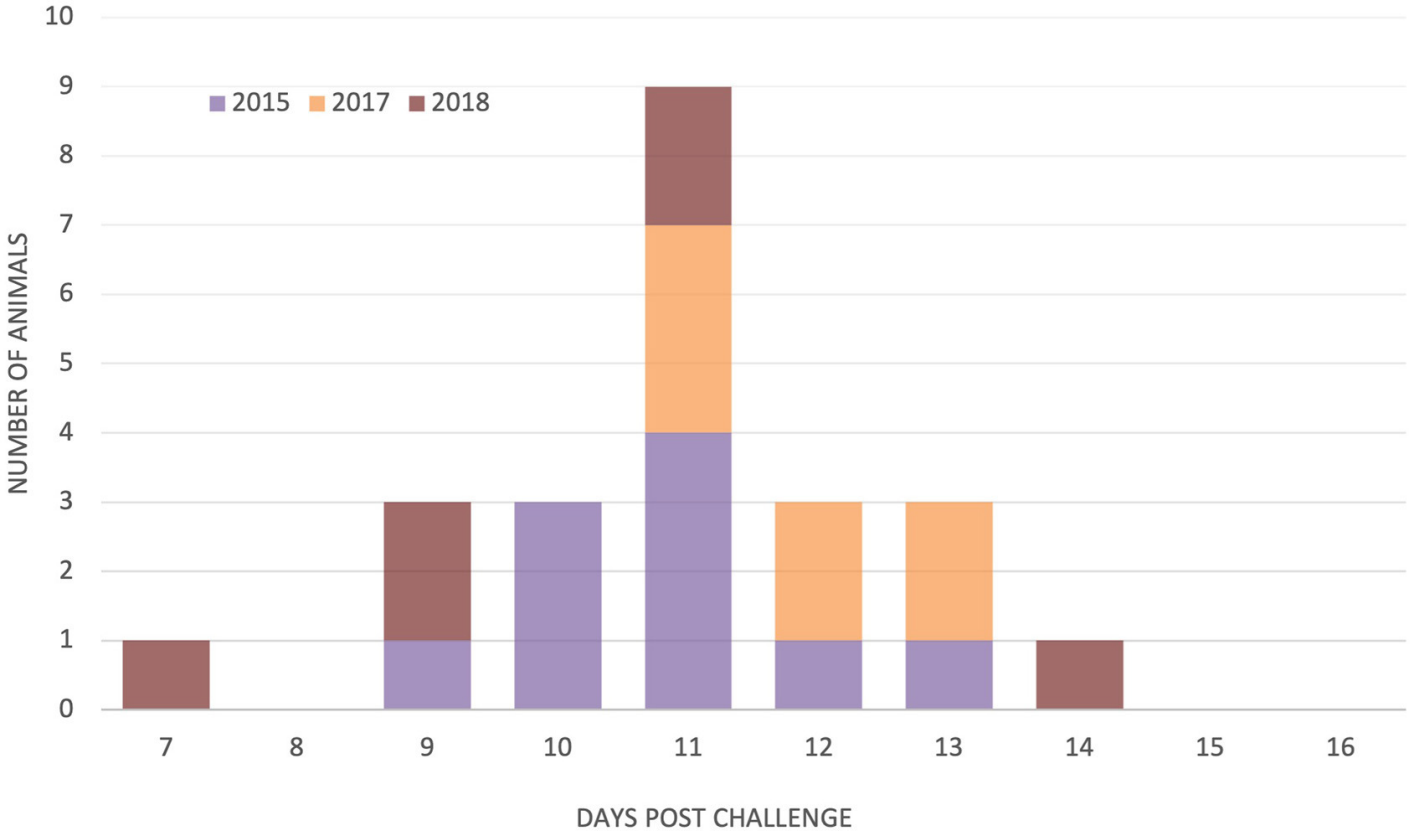
Day of detection of parasitosis in parotid and/or pre-scapular lymph nodes in study animals from three trials *N* = 10 (2015), 7 (2017), and 6 (2018).

Schizonts were detected in the regional lymph nodes of 45 animals. The one animal that did not have detectable schizonts in lymph nodes before death nevertheless developed pyrexia on day 14 was seropositive by day 15, and schizonts were observed on impression smears from organs after death on day 16. The onset of parasitosis was recorded for cattle in the 2015, 2017, and 2018 studies, but it was difficult to accurately determine the first day of lymph node positivity for the 2013 and 2014 studies because sampling occurred every other day. The first day of parasitosis was reasonably uniform for the 2015, 2017, and 2018 studies with no significant difference between the day of onset [*F*_(2, 20)_ = 2.13, *p* = 0.145], with schizonts detected in the majority of animals (21/23) between days 9 and 13, as shown in Figure 3. There was no significant relationship between the time to parasitosis and the time to death (chi^2^ = 1.12, *p* = 0.291).

Only two animals were positive for piroplasms, one in 2013 (17) and one in 2015 showing piroplasms for only 1 day.

### Pyrexia

As shown in Table 2, several indicators of pyrexia were analyzed. All animals developed pyrexia, with a mean time to pyrexia of 13.7 days (Table 2), and all but three animals were pyretic by day 15 (Supplementary Figure 1). The onset of pyrexia varied between studies [*F*_(4, 41)_ = 3.22, *p* = 0.019] as did the time to peak temperature [*F*_(4, 41)_ = 4.54, *p* = 0.004]. Across all studies, there was a significant relationship between time to pyrexia and time to death (chi^2^ = 4.00, *p* = 0.046), where animals that developed pyrexia earlier also died sooner. There was also a significant relationship between the day on which the peak temperature was observed and the day of death (chi^2^ = 25.81, *p* < 0.001), as animals with an earlier peak temperature died sooner. The mean time from onset of pyrexia to death was 4.8 days (Table 2), indicating a relatively short duration of clinical illness. In studies where parasites were examined every day (2015, 2017, and 2018, *n* = 23), almost every animal (*n* = 19) was observed to have pyrexia after detecting parasites, with an overall mean delay of 1.9 days from detection of schizonts to pyrexia.

An indicator of the severity of the pyretic response, peak temperature, did not vary between studies [*F*_(4, 41)_ = 1.684, *p* = 0.172], and there was a significant relationship between peak temperature and the day of death, as animals with higher absolute peak temperatures died sooner than animals with lower peak temperatures (chi^2^ = 5.573, *p* = 0.018). A further indicator of pyretic response, temperature intensity, did vary between studies [*F*_(4, 41)_ = 5.29, *p* = 0.002] and was significantly associated with days to death (chi^2^ = 5.28, *p* = 0.022).

Fourteen animals showed preterminal hypothermia, in which abnormally low temperatures (<38°C) (28) were recorded in the day immediately before succumbing to the disease.

### Packed Cell Volume

The mean PCV decreased slightly throughout the studies in all years (Supplementary Figure 2), with 22 of 46 animals developing mild clinical anemia (PCV 21–23%) (29). The mean values for the final PCV as a percentage of the day 0 PCV for each study presented in Table 2 also demonstrate the decrease in PCV throughout the studies. There was a significant relationship between the percentage change in PCV and days to death with animals that lived longer having decreased PCV (chi^2^ = 6.79, *p* = 0.009). There was a slight increase in PCV between days 16 and 18 postexposure (Supplementary Figure 2), which may be explained by a number of animals (*n* = 11) with a rising PCV in the days before death, potentially associated with hemoconcentration from dehydration.

### White Blood Cell Counts

The mean WBC counts in the 4 days before deaths were 6,200 and 6,700 × 10^6^ cells/L for 2015 and 2017, respectively (Table 2). The WBCs collected in 2013 and 2014 were not collected every day, so comparisons were not made. In 2015 and 2017, WBC counts in 15 of 16 animals showed a decrease 4 days before death compared with the WBC at day 0 (Supplementary Figure 3).

### Plasma Protein Levels

Total plasma protein was measured in the last three studies (2015, 2017, and 2018). A gradual decrease in plasma protein was observed during the course of the studies (Table 2; Supplementary Figure 4). Almost all animals (21/23) developed clinical hypoproteinemia (TPP <60 g/L), with seven animals severely affected (TPP <50 g/L) (29).

### Anti-*T. parva* Serology

We had previously observed that only one of the nine control animals that died in the 2013 study developed a detectable antibody response to *T. parva* (17). Of the 37 animals in the more recent studies, 19 (51%) developed antibodies to *T. parva* (Supplementary Table 1). The earliest recorded seroconversion occurred by day 7, two additional animals seroconverted by day 11, and 14 animals seroconverted by day 16. The results confirm that *T. parva* was a constituent of the field challenge and that seroconversion is not a universal feature of animals that die of *T. parva* infection.

### Non-Continuous Clinical Signs

**Figure 4:**
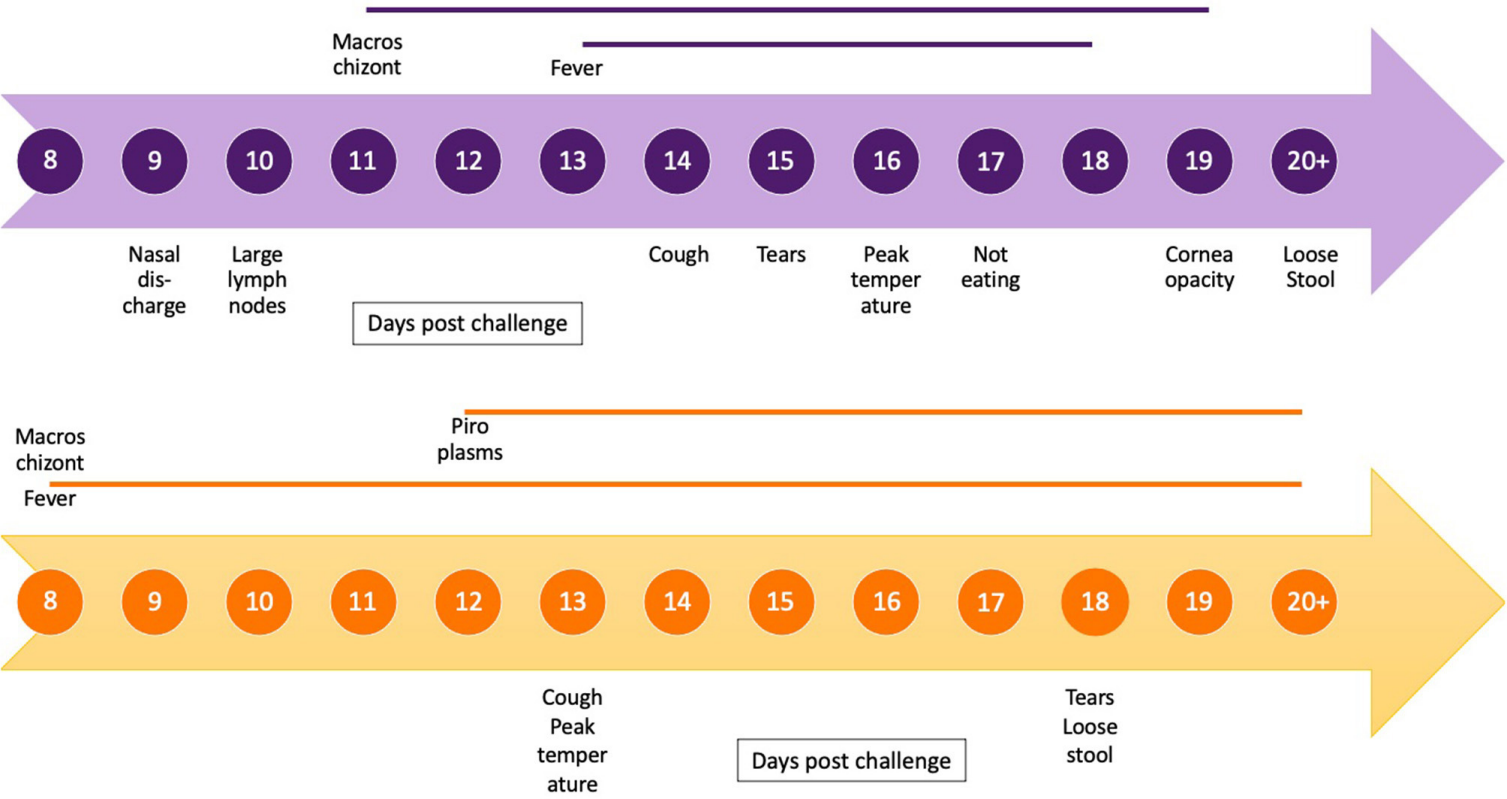
Timeline of clinical progression of corridor disease using mean day post-exposure for onset and duration of clinical signs and presence and duration of macroschizonts in lymph node aspirates (blue) compared with timeline of clinical progression of East Coast fever (orange) as described by Jura and Losos (10).

The frequencies of non-continuous clinical signs observed in each of the four studies are listed in Table 3, and the onset of key clinical signs post-natural exposure is illustrated in Figure 4. The period of clinical disease was very short, with the mean day to death only 9 days after the onset of clinical signs (Figure 4). Figure 4 also includes a graphical representation of the clinical progression of ECF as described by Jura and Losos (10). The experiment described by Jura and Losos was a needle challenge that explains the short incubation period compared with our study. However, the progression of the disease is much longer, as evidenced by the number of days of temperature and macroschizonts.

**Table 3:** Non-continuous clinical signs observed in study animals over five trials *N* = 9 (2013), 14 (2014), 10 (2015), 7 (2017), and 6 (2018).

**Study**	**Corneal opacity**	**Lacrimation**	**Nasal discharge**	**Cough**	**Labored breathing**	**Lymph node swelling**	**Loss of condition**	**Anorexia**	**Neurological signs**	**Teeth grinding**	**Diarrhea**	**Bottle jaw**	**Urticaria**
2013 (*n* = 9)	1	7	9	7	6	9	3	7	0	2	1	1	0
2014 (*n* = 14)	1	11	14	7	8	14	6	7	1	4	0	1	0
2015 (*n* = 10)	1	10	10	10	8	10	4	8	0	6	1	0	2
2017 (*n* = 7)	2	7	7	6	3	7	3	5	0	1	1	0	3
2018 (*n* = 6)	5	6	6	3	4	6	6	4	0	1	2	0	0
Total (*n* = 46)	10	41	46	33	29	46	22	31	1	14	5	2	5

All of the signs, apart from urticaria, have previously been implicated in disease caused by *T. parva* (9, 26). The most consistently observed signs were nasal discharge, swollen lymph nodes, and lacrimation, which were also the earliest signs recorded. Anorexia, labored breathing, and coughing were also observed in more than 50% of the animals. Conversely, the least frequent signs were diarrhea, submandibular edema (“bottle jaw”), and corneal opacity, which developed later in the progression of the disease. One animal in the 2014 study showed neurological signs (staggering upon rising).

### Gross Pathology

The postmortem findings from the 2013 study have previously been described (17). Postmortems were conducted on 28 animals from the 2014, 2015, 2017, and 2018 studies. Necropsy findings were similar in all animals (Supplementary Table 2). Most animals had pulmonary edema (*n* = 27), and many animals had foam within the airways (*n* = 25). The parotid and pre-scapular lymph nodes were enlarged in all animals, and the cut surfaces were edematous and/or hemorrhagic in most cases (*n* = 27).

## Discussion

This manuscript describes in detail the onset and progression of clinical signs and clinical pathology of CD in naturally infected cattle. The additional information from previous reports of CD is a detailed timeline of the evolution of clinical signs of disease, detailed clinical pathology manifestations including changes to PCV, protein and WBC counts, and parasite numbers throughout the disease and performance of diagnostic tests.

The clinical signs of CD were first described by Neitz in 1955 and included fever, enlarged lymph nodes, anorexia, lacrimation, nasal discharge, teeth grinding, loss of condition, coughing, and labored breathing (9). These are also consistent with the signs reported for ECF (13). Uncommon signs in our study were diarrhea, bottle jaw, neurological signs, and corneal opacity, which have been reported as common findings in CD previously (2). The short duration of infection (mean 7.6 days from schizonts to death) might have prevented the development of these more chronic clinical conditions. The difference in disease progression and duration between the CD cases described in our study and a previous study describing ECF are demonstrated in Figure 4 (10), indicating the rapid progression of the disease in our study.

The case fatality in the study animals was 91%. Case fatality to CD in the field in susceptible animals was previously reported to be ~80% (9). The mortality is usually lower for ECF, with case fatality reports for naïve animals under field conditions ranging from 40 to 60% (6, 30). Factors influencing the case fatality in ECF endemic areas include acquired immunity in calves through low but consistent exposure to ticks and low infection rates in ticks resulting from low parasitemias in carrier cattle (31). We demonstrated a high infection rate in ticks (42%) and high abundance and intensity of infected acini per tick (5.95 and 18.8), compared with results reported previously in another study (2.3%, 3.8 and 0.085, respectively) (32). An alternative hypothesis may suggest a virulent strain of buffalo-associated *T. parva* circulating in the study area, as previous research has demonstrated that strains of different virulence will result in different clinical outcomes (18, 33).

There was a significant difference in the time to death between the five studies. This suggests a seasonal effect on tick populations (34). In 2013 and 2018, the studies were conducted in June/July during the colder months, whereas the 2014 and 2015 studies were conducted in November/December and the 2017 study in March during the warmer months. Because the outcome of the disease is dependent on the parasite load, it is possible that seasonal effects reduced the tick population, the challenge and the progression of clinical signs (26).

In contrast, within each study, most animals succumbed within 3 days of each other. Similar observations were made with respect to the time to parasitosis within each study. Previous experimental studies with cattle-derived parasites have shown the time to death, and the detection of parasites in the regional lymph node is strongly dose-dependent, where the parasites were delivered as a sporozoite stabilate (10, 35). Jarrett et al. (36) reported similar results through a detailed analysis of cattle infected by applying infected ticks, although their study did not include a determination of the time to death. These observations suggest that the cattle within each study received a similar dose of parasites.

In our study, the time to parasitosis of 11.0 days (range 7–14) and pyrexia 13.7 days (range 10–19) were similar to previous studies; experimental studies of CD using tick challenge reported the mean time to pyrexia to be 14.5 days (range 7–23) (37). Naturally challenged susceptible animals are expected to show signs of *T. parva* infection 2–3 weeks after exposure (26).

There was no significant relationship between the time to parasitosis and the time to death. Previous studies of ECF have indicated a relationship between lymph node parasitism and the intensity of macroschizonts with the severity of the disease (3). However, in CD, the number of macroschizonts remains low compared with ECF throughout the disease progression and does not relate to the disease severity observed in this study (10). There were significant relationships between the time to death and the time to onset of temperature, peak temperature, and the severity of pyrexia. This is consistent with previous studies that have demonstrated that earlier onset (time to pyrexia) and severity of pyrexia (temperature intensity) are associated with severe disease (3). The peak temperature value and the days to peak temperature have not previously been evaluated in the progression of CD, and these may be explored in the future as potential indicators of the severity of the disease.

Only two animals developed piroplasms, a finding which is consistent with previous studies reporting that CD is self-limiting (there is no carrier state) in the majority of animals (1, 10). This is different from studies of ECF that report piroplasms in animals by day 15 (38). As this was a field study with no control over infections in ticks, we cannot exclude the possibility that the piroplasms observed were of a theilerial species other than *T. parva*.

We demonstrated a slight reduction in PCV over the study period. This is consistent with reports that ECF causes a drop in PCV and mild anemia (38, 39). Anemia in ECF is likely caused by the destruction of RBCs from parasitemia (32). As piroplams were not observed in this study, there may be another mechanism of anemia in CD related to non-regeneration, which may be the result of hematopoietic cell destruction (40). Similarly, we saw a slight reduction in WBC counts, which is consistent with reports of transient leukopenia in fatal cases of CD (2). This is in contrast to studies that have described severe leukopenia in ECF-infected animals (3, 4, 33, 40, 41).

A mild hypoproteinemia was observed in the study animals, which is consistent with studies of ECF (4). This is likely due to the loss of protein in oedematous fluids or may be the result of decreased nutrient intake due to decreased appetite (4). Severe presentations of hypoproteinemia such as “bottle jaw” or subcutaneous edema have been reported to be a common clinical manifestation of CD in other studies but were not observed in this study (2). There may not have been sufficient time to develop these overt clinical signs of hypoproteinemia due to the short duration of infection.

In endemic regions, the diagnosis of ECF or CD is dependent on the presence and severity of clinical signs and should be quickly recognized by cattle owners. The appearance of classical clinical signs, such as nasal discharge, lachrymation, and enlarged lymph nodes, remain the most useful parameters. Confirmatory tests such as lymph node aspirates are effective but may not always be available. We could not compare diagnostic test performance formally due to the small number of animals but determined that ELISA is not an effective diagnostic tool, as it did not detect early cases and not all animals seroconverted. Seroconversion may also occur in asymptomatic animals making its use as a clinical diagnostic tool less valuable. Molecular methods were not assessed in this study and are not likely to be available to cattle keepers. The progression of CD is rapid, so early detection is necessary to implement treatment and prevent lethal outcomes.

Additional to the improved detailed description of clinical CD and associated diagnostic methods, this manuscript also importantly describes the approach for conducting a field experiment for CD in cattle. Apart from seasonal factors outside of our control, we were able to replicate the study over five different occasions with comparable results. The risk with field experiments is ensuring high constant infection pressure for the development of clinical disease in a period amenable to the logistics of maintaining staff at the study site. In this study, we demonstrated that all animals were clinically affected with CD. This enabled a thorough description of the disease progression, including the onset and severity of clinical signs and clinical pathology. To conduct field studies of this nature, there must be trained personnel, including veterinarians, animal health, and laboratory technicians, and adequate, accessible facilities such as a cattle race and crush/squeeze chute. Apart from the research staff, there were two experienced cattle handlers with the study herd 24 h a day. This enabled continual observation of the clinical progression of disease in the study animals and accurate estimates of the onset of clinical signs. The study site was located within Ol Pejeta Conservancy, where the research team had access to a basic laboratory. However, there were challenges regarding electricity that was not continuous, and we required backup batteries to ensure a regular supply and accessibility of the field site, particularly during the 2013 study because of heavy rains. Planning for these obstacles should be considered in advance of any field experiment to avoid wasting resources.

## Data Availability Statement

The raw data supporting the conclusions of this article will be made available by the authors, without undue reservation.

## Ethics Statement

The animal study was reviewed and approved by International Livestock Research Institute Institutional Animal Care and Use Committee.

## Author Contributions

WM and PT conceived the study and secured funding. EC, TS, EP, DW, JP, and PT were involved in field trial design. EC, TS, MC, GN, SM, RA, GP, PL, and PT conducted clinical observations and sampling. EC, TS, MC, GN, SM, and PL carried out laboratory work in the field. MC, PL, and AM processed DNA and RNA extractions. EC and JP performed statistical analyses. EC, TS, and PT wrote the manuscript with feedback from all coauthors. All authors approved submission of the article.

## Funding

This research was conducted as part of the Consultative Group for International Agricultural Research (CGIAR) Research Program on Livestock. Contributors to the CGIAR Trust Fund support ILRI. CGIAR is a global research partnership for a food-secure future. Its science is carried out by 15 research centers in close collaboration with hundreds of partners across the globe (www.cgiar.org). Some of the work described in this paper was supported by grant BB/H009515/1 awarded jointly by the then United Kingdom (UK) Department for International Development and the UK Biotechnology and Biological Sciences Research Council under the Combating Infectious Diseases of Livestock for International Development program. This research was funded in part by the Bill & Melinda Gates Foundation and with UK aid from the UK Foreign, Commonwealth, and Development Office (Grant Agreement OPP1127286) under the auspices of the Centre for Tropical Livestock Genetics and Health, established jointly by the University of Edinburgh, Scotland's Rural College, and the ILRI. This work was also supported by funding from the Biotechnology and Biological Sciences Research Council (BBS/E/D/30002275).

## Author Disclaimer

The findings and conclusions contained within are those of the authors and do not necessarily reflect positions or policies of the Bill & Melinda Gates Foundation nor the UK Government.

## Conflict of Interest

The authors declare that the research was conducted in the absence of any commercial or financial relationships that could be construed as a potential conflict of interest.

## Publisher's Note

All claims expressed in this article are solely those of the authors and do not necessarily represent those of their affiliated organizations, or those of the publisher, the editors and the reviewers. Any product that may be evaluated in this article, or claim that may be made by its manufacturer, is not guaranteed or endorsed by the publisher.
