# Supplementary material for: Clinical Evaluation of Corridor Disease in Bos indicus (Boran) Cattle Naturally Infected With Buffalo-Derived Theileria parva
Source: Front Vet Sci. 2021 Sep 29;8:731238. doi: 10.3389/fvets.2021.731238 (PMC8511504; doi:10.3389/fvets.2021.731238)
Supplement: Supplementary file 1 [file Data_Sheet_1.docx]

Supplementary Files

**Clinical evaluation of Corridor disease in *Bos indicus* (Boran) cattle naturally infected with buffalo-derived *Theileria parva***


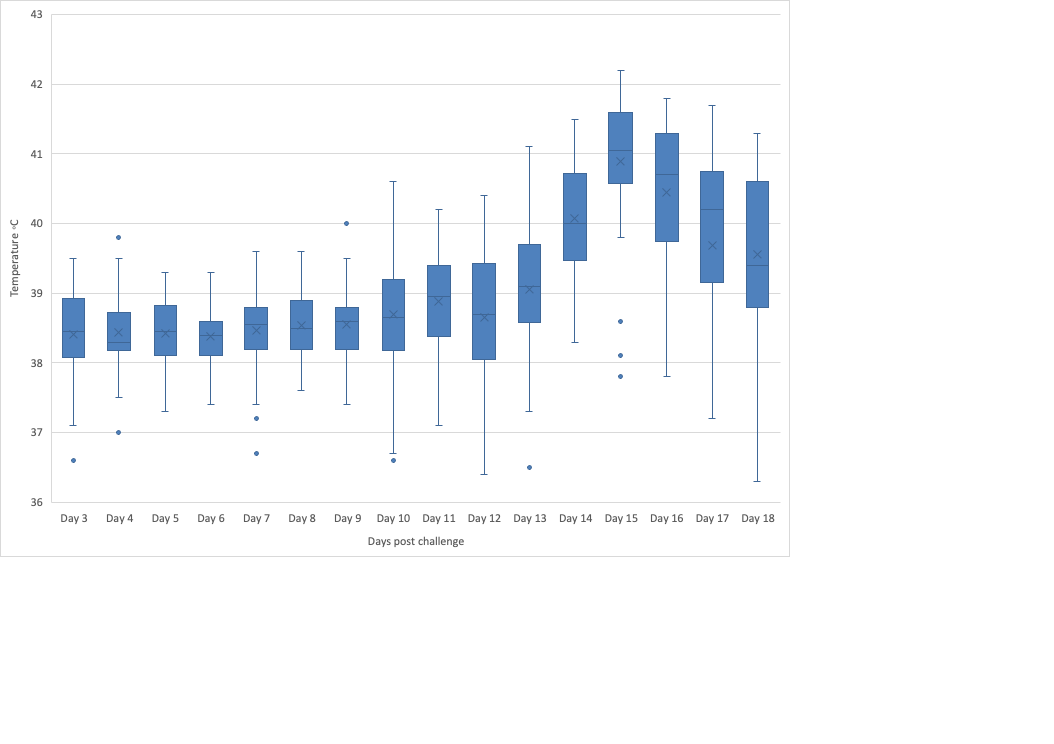
**Supplementary Figure 1.** Box and whisker plots of temperatures of animals in all five studies. N = 9 (2013), 14 (2014), 10 (2015), 7 (2017), 6 (2018). Animals were considered pyrexic when presenting with a temperature over 39.4°C.


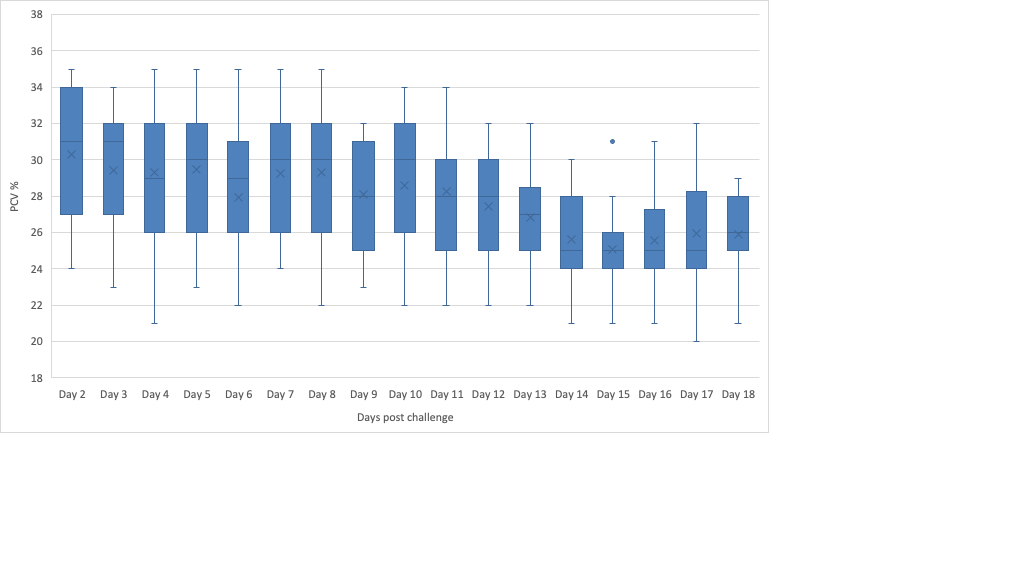


**Supplementary Figure 2.** Box and whisker plots of packed cell volume (PCV) of animals in three trials.

N = 10 (2015), 7 (2017), 6 (2018).


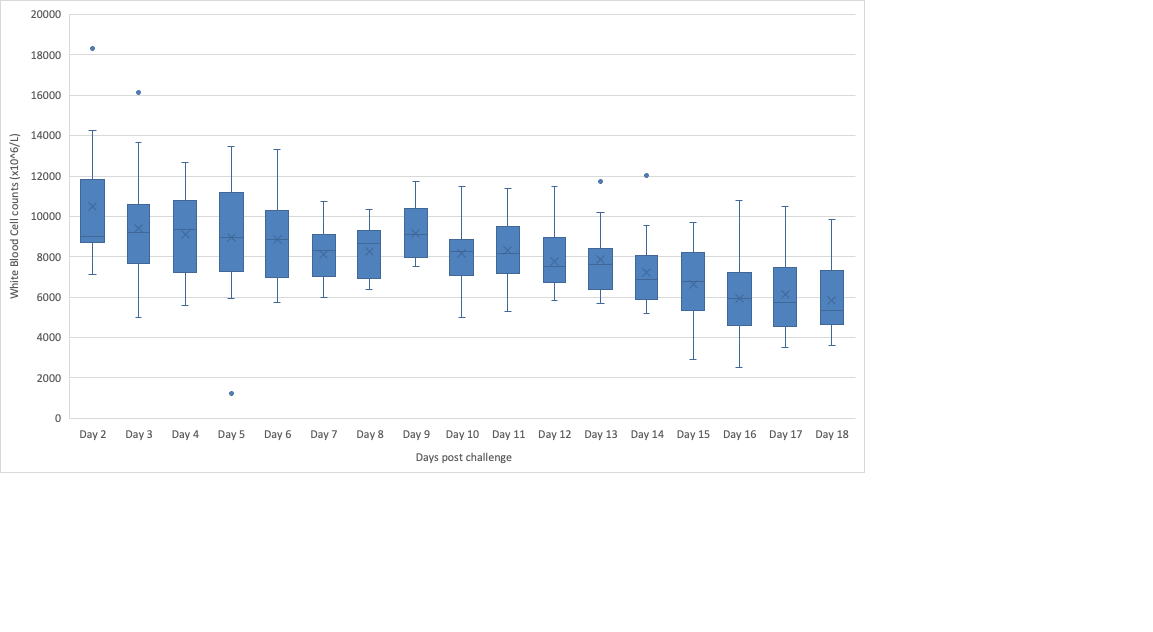


**Supplementary Figure 3.** Box and whisker plots of mean white blood cell count (WBC) in the four days before death for 2 trials. N = 10 (2015), 7 (2017).


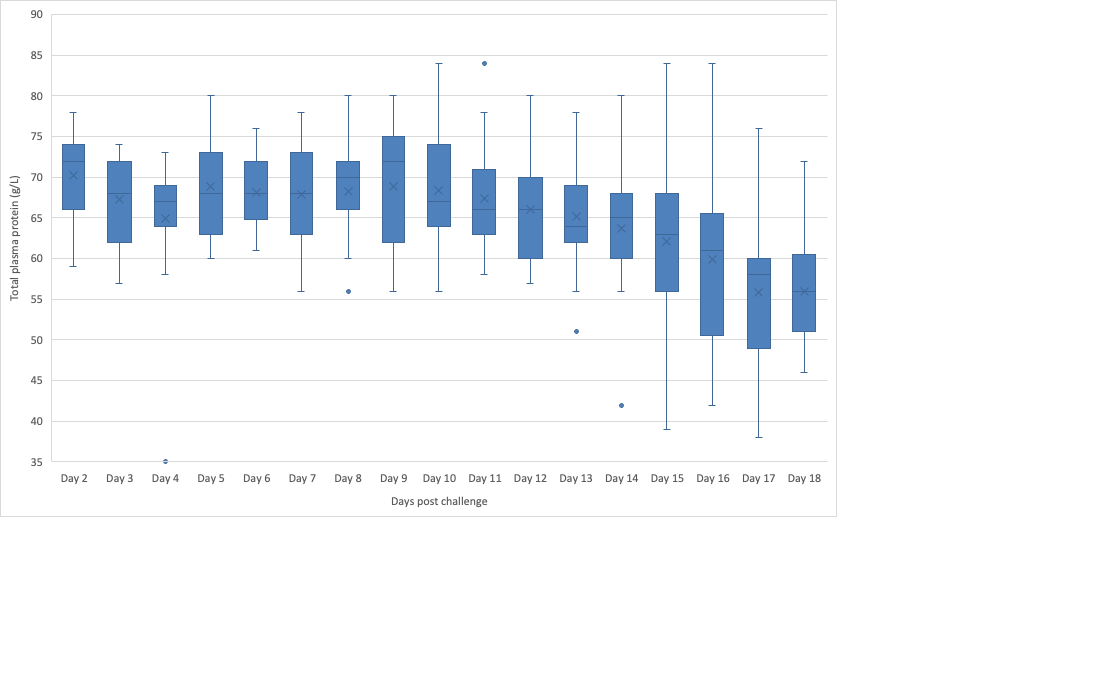


**Supplementary Figure 4**. Box and whisker plots of total plasma protein (TPP) for three trials N= 10 (2015), 7 (2017), 6 (2018).

**Supplementary Table 1**: ELISA values for cattle at five day intervals post exposure. Red highlighted cells indicate positive values. Grey highlighted cells indicate animals had succumbed to disease.

| Animal ID | Pretrial | Day 2/3 | Day 6/7 | Day 10/11 | Day 15 | Day 20 | Day 25/27 |
| --- | --- | --- | --- | --- | --- | --- | --- |
| 2656 | 4 | 3 | 4 | 4 | **24** |  |  |
| 2684 | 16 | 10 | 11 | 13 | 18 |  |  |
| 2689 | 9 | 7 | 8 | 13 | **32** |  |  |
| 2715 | 8 | 2 | 4 | 4 | 6 |  |  |
| 2732 | 18 | 4 | 7 | **50** | **63** |  |  |
| 2740 | 8 | 5 | 6 | 12 | 16 |  |  |
| 2743 | 8 | 6 | 8 | 7 | 16 |  |  |
| 2746 | 9 | 2 | 3 | 3 | 14 |  |  |
| 2754 | 8 | 5 | 8 | 10 | **50** |  |  |
| 2773 | 10 | 4 | 7 | 6 | **22** |  |  |
| 2777 | 5 | 4 | 7 | 10 | 16 |  |  |
| 2767 | 6 | 6 | 4 | 4 | **29** | **68** |  |
| 2637 | 6 | 4 | 4 | 11 | **41** |  |  |
| 2671 | 18 | 5 | 6 | 17 | **50** |  |  |
| 3338 | 6 | NA | 4 | 4 | 7 | 9 |  |
| 3340 | 3 | NA | 4 | 5 | 3 |  |  |
| 3291 | 7 | NA | 6 | 7 | **59** | **80** | **80** |
| 3265 | 15 | NA | 7 | 8 | **23** |  |  |
| 3214 | 18 | NA | **29** | **25** | **26** |  |  |
| 3324 | 8 | NA | 6 | 7 | 13 | 13 |  |
| 3327 | 16 | NA | 9 | 8 | 11 |  |  |
| 3301 | 5 | NA | 7 | 4 | 6 | **33** |  |
| 3247 | 14 | NA | 12 | 9 | **32** | **38** | **22** |
| 3314 | 9 | NA | 10 | 9 | 13 | **29** |  |
| 4124 | 6 | 15 | NA | 15 | 20 |  |  |
| 4218 | 8 | 9 | NA | 10 | 18 | **25** |  |
| 4113 | 8 | 6 | NA | 9 | 16 |  |  |
| 4204 | 6 | 4 | NA | 4 | 7 |  |  |
| 4268 | 6 | 5 | NA | 5 | **26** |  |  |
| 4146 | 14 | 12 | NA | 19 | **24** | **30** | **30** |
| 4142 | 6 | 8 | NA | 8 | 12 |  |  |
| 4807 | 5 | 4 | 4 | 4 | 4 | 4 | 4 |
| 4808 | 8 | 3 | 5 | 6 | 11 |  |  |
| 4795 | 14 | 10 | 9 | 10 | 16 |  |  |
| 4994 | 8 | 3 | 5 | 5 | 22 |  |  |
| 5029 | 6 | 4 | 3 | 4 | 35 | 50 | 75 |
| 4774 | 11 | 6 | 5 | 5 | 7 | 6 |  |

NA = not collected

**Supplementary Table 2**: Gross lesions observed during post mortems of cattle from four trials 2014, 2015, 2017 and 2018

| Animal ID | Foam in airways | Pulmonary oedema | Lymph node oedema | Lymph node haemorrhage |
| --- | --- | --- | --- | --- |
| 2656 | + | + | + | ++ |
| 2684 | ++ | ++ | ++ | ++ |
| 2689 | ++ | ++ | ++ | ++ |
| 2715 | ++ | ++ | ++ | ++ |
| 2732 | + | - | + | - |
| 2740 | ++ | ++ | ++ | + |
| 2743 | ++ | ++ | ++ | + |
| 2746 | ++ | ++ | + | + |
| 2754 | ++ | ++ | ++ | ++ |
| 2773 | ++ | ++ | ++ | ++ |
| 2777 | - | + | + | - |
| 2637 | ++ | + | - | + |
| 2671 | ++ | ++ | ++ | ++ |
| 3338 | ++ | ++ | - | - |
| 3340 | ++ | ++ | + | - |
| 3265 | ++ | ++ | ++ | ++ |
| 3214 | ++ | ++ | ++ | ++ |
| 3324 | - | ++ | ++ | ++ |
| 3327 | ++ | ++ | ++ | ++ |
| 3301 | ++ | + | + | + |
| 3314 | - | ++ | ++ | ++ |
| 4124 | ++ | ++ | + | - |
| 4113 | ++ | ++ | ++ | ++ |
| 4268 | + | + | - | - |
| 4808 | ++ | ++ | ++ | ++ |
| 4795 | ++ | ++ | ++ | ++ |
| 4994 | ++ | ++ | - | - |
| 4774 | ++ | + | + | - |

- absent; + mild; ++ marked
